# Study of the Hydrolytic Stability of Fine-Grained Ceramics Based on Y_2.5_Nd_0.5_Al_5_O_12_ Oxide with a Garnet Structure under Hydrothermal Conditions

**DOI:** 10.3390/ma14092152

**Published:** 2021-04-23

**Authors:** Liudmila Alekseeva, Aleksey Nokhrin, Maksim Boldin, Eugeniy Lantsev, Artem Murashov, Albina Orlova, Vladimir Chuvil’deev

**Affiliations:** Materials Science Department, Physico-Technical Research Institure, Lobachevsky State University of Nizhny Novgorod, 603022 Nizhny Novgorod, Russia; boldin@nifti.unn.ru (M.B.); elancev@nifti.unn.ru (E.L.); aamurashov@nifti.unn.ru (A.M.); albina.orlova@inbox.ru (A.O.); chuvildeev@nifti.unn.ru (V.C.)

**Keywords:** ceramic matrices, immobilization of radioactive wastes, garnet, hydrolytic tests, leaching mechanism

## Abstract

The hydrolytic stability of ceramics based on Y_2.5_Nd_0.5_Al_5_O_12_ oxide with a garnet structure obtained by the spark plasma sintering (SPS) method has been studied. The tests were carried out in distilled water under hydrothermal conditions in an autoclave and, for comparison, in a static mode at room temperature. The mechanism of leaching of Y and Nd from the ceramics was investigated. It has been shown that at “low” temperatures (25 and 100 °C), the destruction of pores occured, and the intensity of the leaching process was limited by the diffusion of ions from the inner part of the sample to the surface. At “high” test temperatures (200 and 300 °C), intense destruction of the ceramic grain boundaries was observed. It was assumed that the accelerated leaching of neodymium is due to the formation of grain-boundary segregations of Nd^3+^ in sintered ceramics.

## 1. Introduction

Handling of high-level waste (HLW) including minor actinides (MA) accumulated over many years of nuclear power operation is an important task of modern radiochemistry. The high-level waste generated after the extraction of uranium and plutonium from spent reactor fuel is known to contain radioactive isotopes of about 40 elements, 27% of which are the Rare Earth Elements (REE). The study of such compounds is very important for the immobilization of actinide elements (which constitute a special group of long-term ecologically hazardous radionuclides) in order to isolate these ones from the biosphere for the long time required for the storage and disposal. For this purpose, the world’s leading laboratories are currently studying ceramic materials based on natural minerals [1]: monazite [2,3], garnet [3,4,5,6,7,8,9,10,11,12,13,14,15,16,17], kosnarite [18,19,20,21], pyrochlore [10,22,23,24,25], scheelite [26], etc. The garnet structure is one of the most promising matrices for the MA immobilization.

Garnets belong to a group of minerals with the general formula B^2+^_3_R^3+^_2_(XO_4_)_3_. The garnet structure is stable in a very wide range of cationic substitutions, and in all crystallographic positions. The position of the B^2+^ cation can be occupied by Mg, Fe, Mn, Ca; actinides (including Pu (IV)) and REE; the concentration of the substitutes can achieve 4–16 wt% [5,6]). The position of the R^3+^ cation can be occupied by Al, Fe, Cr, and Ga, the position of the X cation—by Al, Fe, Ga, and Si cations [3,6]. In addition, compounds with a garnet structure have high hydrolytic and radiation stability [3,4,7,8,9,27].

In the present work, the hydrolytic stability of ceramics based on Y_2.5_Nd_0.5_Al_5_O_12_ (YAG/Nd) oxide with a garnet structure obtained by the spark plasma sintering (SPS) method at elevated temperatures and pressures was studied. The SPS process is based on high-rate heating of powders in vacuum or in an inert ambient by simultaneously applying a strong millisecond electric DC pulses and a constant pressure to a graphite mold containing the powder [20,28,29,30,31,32,33,34,35,36,37]. An opportunity to reduce the sintering time drastically (as a compared to conventional sintering process) while preserving a high density of obtained material is an important advantage of SPS process, especially relevant for the radioactive materials. High heating rates in SPS allow limiting the grain growth considerably and provide conditions for the resulting phase composition of the sintered ceramic to remain identical to the one of the initial powders. This affect positive the hydrolytic, thermal, and radiation stability of the produced ceramics. This is true for simple (oxide) compositions as well as for more complex saline ones composed of many kinds of ions. In recent years, the technologies for producing the garnet-based ceramics the developing rapidly and intensively. In [38], electron beam assisted synthesis was proposed for producing the ceramics based on yttrium-aluminum garnet doped with lanthanides. The ceramic production time was ~1 s. However, the photographs presented in [38] indicate a structure inhomogeneity of the ceramics, which can affect its properties negatively.

The mechanisms of high-speed sintering of Y_2.5_Nd_0.5_Al_5_O_12_ ceramics with the garnet structure were described in detail in [3,39,40]. In present work, a special attention was paid to the problem of studying the high-temperature hydrolytic stability of ceramics with a garnet structure. In [11], the hydrolytic stability of various garnet single crystals (including pure yttrium–aluminum garnet not doped with lanthanides) was investigated at 200 °C and a pressure of 150 bar for 28 days. The leaching rate of yttrium ranged from 1.29 × 10^−3^ to 5.64 × 10^−4^ g/m^2^·day. Note that there is almost no information on the hydrolytic resistance of ceramics with the garnet structure at elevated temperatures (200–300 °C). This makes it difficult to predict the long-term hydrolytic resistance of the ceramics obtained by SPS.

## 2. Materials and Methods

The Y_2.5_Nd_0.5_Al_5_O_12_ garnet powder was obtained by the co-precipitation method. An aqueous solution containing Y(NO_3_)_3_, Nd(NO_3_)_3_, and Al(NO_3_)_3_ was mixed with a 5% ammonia solution. The resulting mixture was heated for dehydration up to 90 °C. The dry residue was heated at 300, 500, 800, and 1000 °C for 6 h at each stage and subjected to dispersing in an agate mortar after each thermostating (annealing) stage.

The ceramics were obtained by the SPS method on a Dr. Sinter^®^ Model-625 system (NJS Co., Ltd., Tokyo, Japan). The powders were placed in a graphite mold with the inner diameter of 12 mm and heated by passing the of high power millisecond electric current pulses (up to 3 kA). The sintering temperature was 1400 °C. It was measured with a Chino^®^ IR-AH pyrometer (Chino IR-AHS2 infrared, Chino Corporation, Tokyo, Japan) focused onto the graphite mold surface. Sintering was carried out in vacuum (6 Pa). The heating rate was 50 °C/min, the uniaxial pressure applied was 70 MPa. The uncertainly of the temperature measurements was ±10 °C, the one of the pressure maintenance was 1 MPa. The density of the obtained ceramics was measured by hydrostatic weighing in distilled water on a Sartorius^®^ CPA balance (Göttingen, Germany).

The phase composition of the obtained ceramics was studied by X-ray diffraction (XRD) method using Shimadzu^®^ LabX XRD-6000 diffractometer (Shimadzu, Kyoto, Japan) with a Cu K_α_-filter (the emission wavelength λ = 1.54078 Å) in the diffraction angler range of 2θ = 20–60° with the step 0.02° and exposure 0.6 s at every point. The structure of the ceramics was studied with a Jeol JSM-6490 scanning electron microscope (SEM, Jeol Ltd., Tokyo, Japan) with an Oxford Instruments INCA 350 energy dispersive spectrometer (EDS, Oxford Instruments pls., Oxford, England). The average size of the grains and particles of the second phase was determined with GoodGrains 2.0 software. The specimens for the grain-structure study were mechanically polished with diamond suspension and finished to roughness under 1 μm.

The hydrolytic stability of ceramics was studied under hydrothermal conditions in an autoclave at temperatures of 100, 200, and 300 °C in distilled water. The tests were carried out in a metal autoclave with an internal volume of 3000 cm^3^. the ceramic samples were placed in ceramic beakers placed inside the autoclave (the volume of the beakers (100 cm^3^) was much less than the one of the autoclaves). The uncertainty of maintaining the temperature was 5 °C. The tests were carried out at temperatures of 100 °C (pressure 0.1 MPa), 200 °C (1.56 MPa), and 300 °C (8.59 MPa). Thus, the tests were carried out according to the scheme: Stage 1: 100 h, Stage 2: +200 h, Stage 3: +200 h, Stage 4: +200 h, Stage 5: +300 h. For comparison, leaching was also carried out in a static mode at room temperature (25 °C).

Water samples for testing were taken after testing, for a total time of 100 h, 300 h, 500 h, 700 h, and 1000 h. The concentration of elements passed into the solution during leaching was determined by inductively-coupled plasma mass spectrometry (ICP-MS) using Thermo Scientific^®^ ELEMENT^TM^ 2 high-resolution mass spectrometer (Thermo Scientific, Bremen, Germany) within the framework of an external calibration. The calibration was carried out using solutions in the ICP-MS-68A-A multielement standard (High Purify Standards, North Charleston, SC, USA). Before the experiment, the ceramic samples surfaces were mechanically grinded and polished to the roughness level Rz20 in order to remove graphite residues.

To calculate the leaching rate of the cations, the weight losses of the component *i* (normalized weight loss) were calculated using the formula:

*NL_i_ = a_ki_ / (M_oi_ × S)*
, (1)

where *NL_i_* is the normalized weight loss of the *i*th component (in g/cm^2^), *a_ki_* is the net weight of the *i*th component passed into the solution during leaching (in g), *M_0i_* is the mass concentration of the *i*th *I* component in the sample at the beginning of tests (in g/g), and *S* is the open area of the sample surface (in cm^2^).

The leaching rate of the *i*th component *R_i_* was calculated according to the formula:

*R_i_ = NL_i_ / t_n_*, (2)

where *t_n_* represents the time interval (in days).

The leaching mechanism for Y and Nd from investigated ceramics was evaluated in accordance with the de Groot-van der Sloot model [41], which can be represented as an equation of a linear dependence:

lg*B_i_* = Algt + *const*, (3)

where *B_i_* is the total yield of the *i*th element from the sample during the time of contact with water (in mg/m^2^), and *t* is the contact time (in days).

The quantities *B_i_* were calculated using Equation (4):

*B_in_ = C_in_ (L/S)√t_n_/(√t_n_ – √t_n–1_)*, (4)

where *C_in_* is the concentration of *i*th element in the leaching solution by the end of *n*th period (in mg/L); *L/S* is the ratio of the solution volume to the sample surface area (in L/m^2^); *t_n_* and *t_n_*_–*1*_ are total contact times at the end of *n*th period and its beginning, respectively (in days).

## 3. Results and Discussion

**Figure 1:**
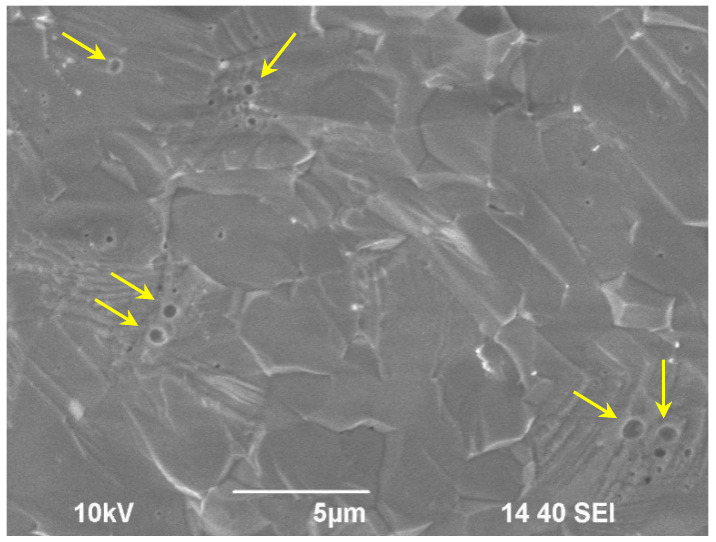
SEM image of a fracture of a sintered ceramic sample.

The powders obtained were pale violet colored with a polycrystalline sample. The composites obtained contain easily destructible agglomerates ranging in size from ~1 to ~10 µm. The sintered ceramics had a dense fine-grained microstructure with an average grain size of 3–10 µm (Figure 1). The relative density of the sintered samples was ~99.5–99.7% of the theoretical value (*ρ_th_* = 4.77 g/cm^3^). There were no microcracks on the surface of the sintered samples. Few pores of micron and submicron sizes were observed on the fracture of the sample (see, for example, Figure 1; the large pores are marked with yellow arrows).

**Figure 2:**
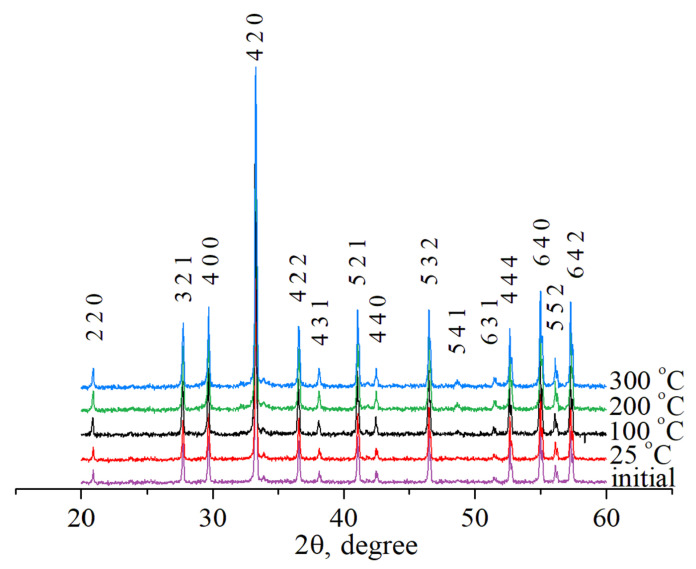
XRD curves for ceramics Y_2.5_Nd_0.5_Al_5_O_12_ before (initial) and after leaching.

The ceramics were identified to be single phase materials with a garnet structure (sp. gr. Ia3d, ICDD Card No. 08-0178). There were no changes in the phase composition during leaching (Figure 2).

**Figure 3:**
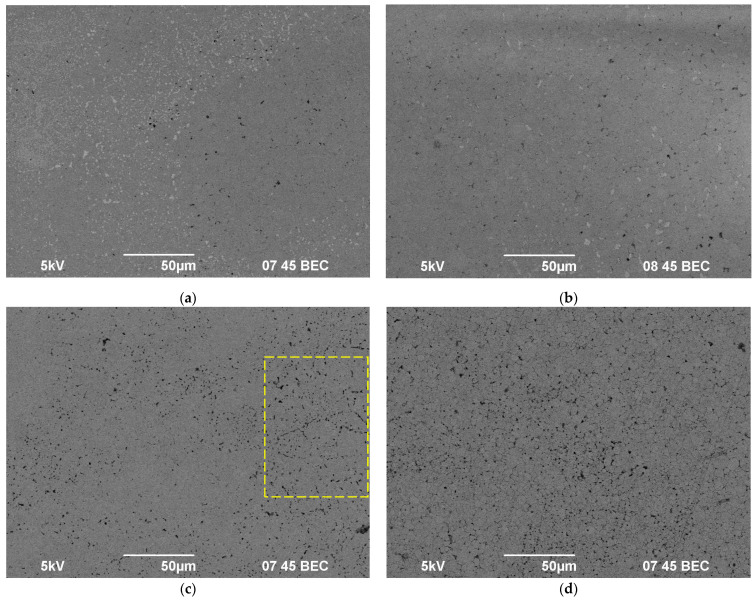
SEM images of the ceramic samples surface after hydrolytic tests for 1000 h at room temperature (**a**) at 100 °C (**b**) at 200 °C (**c**) and at 300 °C (**d**).

The study of the microstructure of the samples after the leaching tests has shown the test temperature to affect the nature of the surface destruction significantly. At “low” test temperatures (25 and 100 °C), a destruction of the surface near the pores is observed (Figure 3a,b). Some light deposits were observed on the surface of the samples. Most likely, these ones were formed as a result of the interaction of the test environment with the materials of the autoclave. Holding in distilled water at a 200 °C for 1000 h led to a drastic increase in the etching intensity of the sample surface (Figure 3c). It is important to note that the destruction areas on the sample surface were arranged along the grain boundaries (in Figure 3c, such an area is framed by a yellow dashed rectangle). 

**Figure 4:**
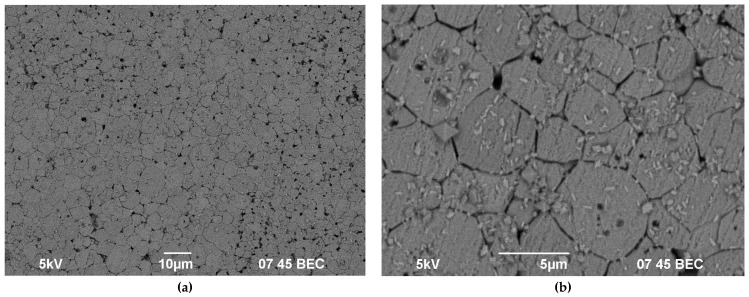
Surface of a ceramic sample after hydrolytic tests for 1000 h at a temperature of 300 °C (magnification: (**a**) ×1000, (**b**) ×10,000).

With further increase of the test temperature up to 300 °C, the destruction intensity at grain boundaries increased. After 1000 h of testing, the entire surface of the sample was covered by traces of the “intergranular corrosion” (Figure 3d and Figure 4a). Thus, the mechanisms of surface destruction at “low” and “high” test temperatures differ significantly. At “low” temperatures, the destruction is observed mainly near the pores whereas at “high” temperatures the destruction of ceramic grain boundaries took place. We assume that there are two factors promoting accelerated destruction of grain boundaries in hot water: (i) the special composition of the ceramic grain boundaries; (ii) special chemical reactions of water with ceramics at elevated temperatures leading to “intergranular corrosion”. In our opinion, both of these factors are important since “intergranular corrosion” was observed at elevated temperatures only. At present, it is difficult to identify which of these two factors is most important.

Probably, one of the factors promoting the accelerated destruction of grain boundaries in YAG/Nd ceramics is segregation of neodymium at these ones. As shown in [42], the grain boundaries of the YAG/Nd ceramics obtained by the high isostatic pressure (HIP) method contained a higher neodymium concentration than the crystal lattice inside the grains. This assumption, according to [43], can explains a number of anomalies in the optical properties of YAG/Nd ceramics.

**Figure 5:**
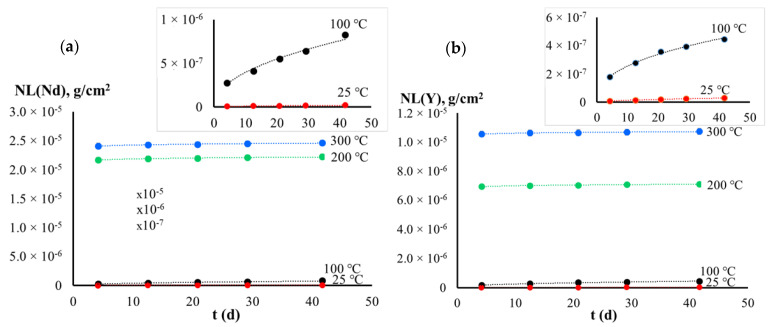
The normalized weight loss *NL_i_ vs* the time *t* for Y (**a**) and Nd (**b**).

**Figure 6:**
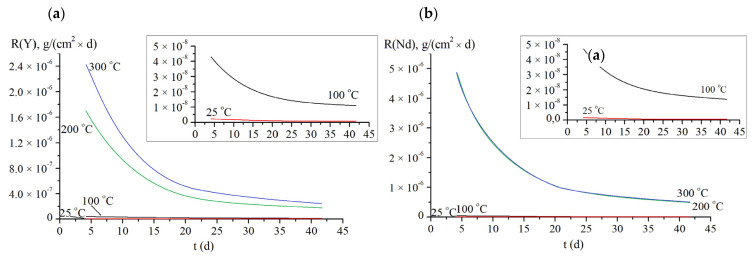
The leaching rate *R_i_* vs the time *t* for Y (**a**) and Nd (**b**).

This conclusion is confirmed by the assessment of the leaching mechanism according to the de Groot and van der Sloot model. The smallest leaching rates of yttrium and neodymium are given in Table 1. The graph of the normalized weight loss and of the leaching rates *R_i_ vs* the time *t* are shown in Figure 5 and Figure 6. As one can see from the data obtained, the leaching rates of cations under hydrothermal conditions were significantly higher than the ones in the static regime at room temperature. Furthermore, it increased with increasing temperature up to 200 °C. However, the leaching rates of cations remained almost unchanged with further increasing test temperature up to 300 °C. The leaching rates achieved characterize the samples studied as having a high hydrolytic stability. The leaching rate of yttrium was comparable to the data obtained for the YAG single crystal [11]. We assume the insignificant differences observed are associated, first of all, with the presence of residual porosity as well as with more accelerated destruction of grain boundaries in YAG/Nd ceramics sintered. Because of the low leach rates measured and the flexibility of various garnet structures to incorporate a wide range of REEs, the investigated garnet are promising as viable waste forms for hosting minor actinides from nuclear wastes.

The analysis of the data presented in Table 1 shows that at elevated temperatures (100–300 °C) the rate of neodymium leaching is higher than the one for yttrium. This cannot be due to differences in the diffusion coefficients of Y and Nd in the YAG crystal lattice. According to [44], the effective diffusion coefficient of Nd^3+^ in fine-grained YAG ceramics can be calculated using the equation: *D_Nd_* = 177 × exp(−623 [kJ/mol]/*RT*) (in m^2^/s). In [45,46], it was reported that the diffusion activation energy and diffusion coefficients of Nd^3+^ in YAG/Nd ceramics are close. The activation energy of diffusion of Y^3+^ in YIG (yttrium–iron garnet) is ~502 kJ/mol, and the diffusion coefficient of Y in YIG garnet is much higher than the one of Nd in YAG [45]. In [47], the highest values of the diffusion coefficient of Y^3+^ in YAG (2.43 × 10^−10^ cm^2^/s at 1750 °C) were reported. It [48], the activation energy of grain boundary diffusion of yttrium Y^3+^ for YAG garnet was reported to be ~565 kJ/mol. It is less than the activation energy of grain boundary diffusion of Nd^3+^ (~637 kJ/mol). Also, a low activation energy for Y^3+^ diffusion in the YAG lattice was reported to be ~530 kJ/mol [49].

Thus, the accelerated leaching of Nd observed in the sintered ceramics cannot be explained by differences in the diffusion coefficients of Nd^3+^ and Y^3+^ ions: the analysis of the literature shows that the diffusion coefficient of Y^3+^ in YAG exceeds the one of Nd^3+^. In our opinion, the leaching of neodymium observed during the hydrolytic tests is due to an increased segregation of Nd^3+^ at the grain boundaries of garnet (see above).

**Figure 7:**
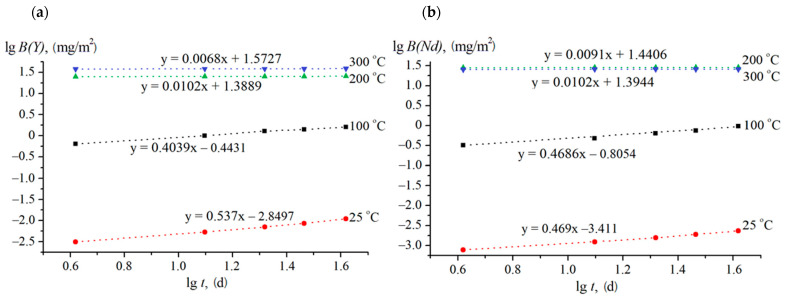
Logarithmic dependence of Y (**a**) and Nd (**b**) yield on the time of contact with water.

To clarify the mechanism of leaching of Y and Nd from the ceramics, the de Groot and van der Sloot dependence were plotted (Figure 7). The slopes of the approximating straightline point to the predominant leaching mechanism. Earlier, the values of coefficient A in Equation (3) were shown to point to the following leaching mechanisms: A < 0.35—leaching from the surface; A = 0.35–0.65—diffusion from inner layers; A > 0.65—dissolution of the surface layer [50,51]. The leaching of Y and Nd in the static mode and under hydrothermal conditions at 100 °C was found to be due to diffusion from the inner layers of the ceramic (A = 0.4–0.54, Figure 7). At 200 and 300 °C under hydrothermal conditions, leaching from the ceramic surface took place (A ~0.01, Figure 7).

In our opinion, a change in the destruction mechanism of the samples surface during hydrolytic tests is one of possible origins for the change in the leaching mechanism at “high” test temperatures (see Figure 3 and Figure 4 ). The grain boundaries of polycrystalline materials are known to have an increased diffusion permeability—the grain boundary diffusion coefficient in fine-grained materials is much higher than the one in the crystal lattice [52,53]. This suggests the diffusion of diffusion of Y and Nd ions to the surface to occurs mainly along the grain boundaries.

In conclusion, it should be noted that such an intergranular nature of fracture may be undesirable, since the crack-like defects formed on the surface can be sources of the premature destruction of ceramics under thermal shock (rapid temperature changes) or under mechanical stress.

## Figures and Tables

**Table 1:** Minimum achieved leaching rate of cations.

Experimental Conditions	T (°C)	Leaching Rate *R_i_* (at 1000 h), g/(cm^2^·d)	
		Y	Nd
Static Mode	25	7.11 × 10^−10^	4.14 × 10^−10^
Hydrothermal Conditions	100	1.08 × 10^−8^	1.38 × 10^−8^
	200	1.75 × 10^−7^	4.99 × 10^−7^
	300	2.46 × 10^−7^	4.97 × 10^−7^

## Data Availability

Data is contained within the article.
